# This Bloke Who Helps Me With My Tractor, He’s Been the Best Psychologist: The Experience of Seeking Mental Health Support in Rural Australia

**DOI:** 10.1177/15579883241249103

**Published:** 2024-04-30

**Authors:** Brenda Mutsvairo, Daniel Terry, Blake Peck

**Affiliations:** 1 School of Nursing and Midwifery, University of Southern Queensland, Ipswich, Queensland, Australia; 2 Institute of Health and Wellbeing, Federation University, Mt Helen, Victoria, Australia; 3 Centre for Health Research, University of Southern Queensland, Ipswich, Queensland, Australia

**Keywords:** health care utilization, health care issues, mental health, psychosocial and cultural issues, qualitative research, research

## Abstract

Mental illness is difficult to discuss among men due to notions of remaining tough, being a man, and societal expectations. In rural communities this is particularly evident which is further exacerbated by poor health care access. The aim of this study is to understand the lived experiences of men and their significant others when seeking mental health support in rural areas. A qualitative study was conducted using purposeful sampling. Data were collected using semi-structured interviews in rural or regional areas of Australia. Open-ended questions were asked but more questions were developed from the responses given. Data analysis was conducted using thematic analysis. Four key themes emerged. These encompassed triggers and help-seeking caused by stressors such as work, family, and poor physical health, with support seeking from professional or informal supports. The second theme included challenges securing professional support appointments, while the third was centered on access to medication and travel time. Finally, the final theme encompassed relationships being impacted by poor mental health or created insights into the need to seek help. The experiences explored throughout this study highlight that as men are impacted, so too are married or romantic partners and children; however, they are the catalyst for help-seeking. The study further highlights even when men are psychologically prepared to seek help, it may be difficult to do so. Improving access goes beyond mere medical professionals in rural areas and must focus on supporting families and loved ones to support men.

## Introduction

Mental health conditions are quite challenging due to stigma, masculinity, stoicism, access, and low health literacy have been identified to minimize access to mental health support for men in rural or regional areas of Australia (Fitzpatrick et al., 2021; Schlichthorst et al., 2016). According to Stormacq et al. (2020), health literacy is defined as an individual’s ability to understand, assess, access, and apply health information to enable making reasonable health decisions. Furthermore, in this context, mental health literacy is defined as beliefs and knowledge about disorders of mental health, which help their identification, management, and prevention (DeBate et al., 2022). It is identified as a challenge for men, while being a barrier to seeking help (DeBate et al., 2022).

In Australia, approximately 20% of the community has reported having a mental health condition, which is similar to the proportion observed internationally (Affleck et al., 2018). However, Dolja-Gore et al. (2018) have reported that 18% of Australian men experience or continue to experience poor mental health, which is suggested by Ferrari et al. (2022) to be higher than the global average among males of 11.9% (range 11.0%–12.9%). Owing to factors such as stigma and masculinity there is a propensity for men to under-report poor mental health (Ellis et al., 2013; King et al., 2020). Thomas et al. (2015) suggested that there are additional complexities in Australia due to challenges associated with rurality and accessing the requisite services in a timely manner. These complexities are particularly evident among men due to a variety of triggers which include long working hours, climate change, life stressors, social isolation, and physical illness (Brew et al., 2016; Kaukiainen & Kõlves, 2020; Schlichthorst et al., 2016). Examples of these complexities include poor use of health services, poor quality of life, and access to services (DeBate et al., 2022; Kaukiainen & Kõlves, 2020).

In addition to these triggers, challenges such as stigma, masculinity, stoicism, access, and low health literacy not only lead to under-reported but have been identified to disrupt men accessing mental health support, particularly in rural or regional areas of Australia (Fitzpatrick et al., 2021; Schlichthorst et al., 2016). Beyond under-reporting or health seeking, Hiebert et al. (2018) have indicated that men’s higher rates of suicide may also occur due to the stigma associated with mental illness. In Australia, the suicide rates in rural areas in 2022 were 17.1 per 100,000 in regional areas, 23.7 per 100,000 in remote areas, and 24.5 per 100,000 in very remote areas (Australian Institute of Health and Welfare, 2024).

These findings are further supported by research in other countries where under-reporting occurs among men; they are less likely to want to acknowledge depressive symptoms and are directly related to levels of masculinity (Affleck et al., 2018). This may exacerbate the propensity of suicide among men, who may see this as a solution due to the challenges of no one to talk to and the fear of being judged by others; however, some men may be prepared to share their experiences of depression (Brownhill et al., 2002). Nevertheless, Fogarty et al. (2015) reported that this preparedness to talk and share is inhibited by either the discouraging responses of other people or by cultural expectations in which men find themselves that valorizes strength and stigmatizes emotional expressiveness. The underutilization of health services is similar to Australia, where stoicism, masculinity, and the need to self-manage poor mental health are perpetuated (Affleck et al., 2018). Therefore, McKenzie et al. (2022) reported that the relationship between stigma and masculinity limits men’s disclosure of their mental health challenges and opportunities to obtain social support, thus creating a barrier to seeking help and obstructing adherence to treatment.

Stoic masculinity and gender norms have been reported to be more prevalent in rural and specifically farming communities, where male-dominant occupations and social norms may impact help-seeking behaviors (Perceval et al., 2018). For example, when men encounter challenges, they are expected to “man up” and deal with the situation, and emotions are suppressed due to fear of being judged as “not man enough.” Apart from stoicism and suicide in rural areas, limited access to health care in rural areas is another key factor why men are reluctant to seek help (Perceval et al., 2018). In some cases, health services are just not available due to inequities in health care (Thomas et al., 2015). The unavailability of services is due to the geographical location and lack of specialized staff such as psychologists or other mental health professionals, which limits access as individuals must travel long distances to seek help, as highlighted by Kaukiainen and Kõlves (2020).

Beyond the various factors that impact the mental health of men and impede their health seeking, significant others and family dynamics are also impacted by men’s mental illness (Kamis, 2020; Lawn & McMahon, 2014). Significant others also experience mental illness themselves due to their proximity to and caring role of men with poor mental health (Lawn & McMahon, 2014). It has been suggested to be related to the stress and anxiety when caring for their loved ones. Mental health within an intimate relationship causes additional stress and strain and can lead to divorce, which then further perpetuates mental illness among both parties and has been reported to lead to suicide among men (Oliffe et al., 2022). Despite the added strain mental illness has on a relationship, it has been identified that men feel comfortable expressing their emotions to their partner, and there is a higher level of persuasion achieved by an intimate partner or loved one in having men seeking mental health support (McCabe et al., 2015).

At present, there are ongoing behaviors among men who delay seeking help, and there remains a dearth of research that explores mental health of men, particularly those living in rural areas (Kilpatrick et al., 2015). In addition, some men continue to be avoidant of health care professionals when it comes to mental health (Hiebert et al., 2018). As such, it is vital to identify the challenges encountered by men regarding their mental health, due to isolation, work, relationship stressors, lack of immediate access to support, further exacerbated by geographical location, financial constraints, and loss of land. Within this context, the aim for this study is to understand the lived experiences of men when seeking mental health support in rural or regional areas and the experience of their significant others.

## Methods

To address the research aims, phenomenological exploratory approach was used to understand the experiences of men and their partners living in rural areas while seeking mental health support. Particularly a focus on barriers and enablers they have encountered. Phenomenology seeks to understand and describe the core of a phenomenon by examining it from the perspective of those who have experienced it (Neubauer et al., 2019). Grossoehme (2014) has indicated that phenomenology seeks to provide the theoretical foundation that allows for the uncovering of meaning that each individual ascribes to their experience.

### Sample

A purposeful sampling approach was employed to identify and select six to eight males who already have or have had a mental health issue but are receiving mental health support and were aged between 25 and 65 years. The inclusion criteria included males with or without partners who lived in the rural or regional areas of Australia, history of mental illness, and significant others. This group was selected due to the gaps identified in literature on the help-seeking patterns in this population. Participants were recruited through known contacts and through social media posts on key men’s mental health social media support network groups. In addition, the sample included a male participant’s significant other, such as a partner, spouse, close relative, or friend. It was vital to invite significant others to participate given they often play an important role for men to seek help (Labra et al., 2019). However, it was not compulsory for the significant other to participate. Men were excluded if they did not have or had a previous mental health issue, were living in a metropolitan area, or were younger than 25 years or older than 65 years of age. The final number of participants consisted of three males and one female, who was the wife of one of the participants. It must be noted the two other male participants’ partners were not willing to participate, while the other was deceased. Finally, two participants lived in rural Victoria, and two were from rural Tasmania.

### Data Collection

Data were collected through one-on-one semi-structured interviews with each participant by one of the researchers who had a professional mental health background. As such, this background and training enabled open-ended questions to be asked to elicit the lived experience of the individual without further impacting their mental health by reliving key experiences. Interview length was approximately 30 to 45 minutes and based on what the preference of the participant was either audio via telephone or video recorded through videoconferencing technology. The semi-structured interview questions included but were not limited to asking participants to discuss their experience when they realized they were having or had a mental health issue. While other questions related to how was a significant other impacted by their partner’s mental health challenges. The semi-structured nature of the questions enabled the examination of key elements that were raised and to uncover key aspects of the lived experience in detail.

### Data Analysis

To analyze the data, thematic analysis was used to capture the themes and patterns of meanings throughout the dataset (Braun & Clarke, 2021; Damayanthi, 2019). The process included the five phases of thematic analysis through familiarization, systematic data coding, generating themes from coding and collating data, reviewing and developing key themes, and finally, refining, defining, and naming themes (Braun & Clarke, 2021). Familiarization was achieved through listening and transcribing, enabling the researcher to fully emerge in the data and what was discussed (Damayanthi, 2019). Second, during the coding process key sections of the text are pinpointed and assigned labels, followed using mind-maps, tables, or arranging codes into “theme-piles” to assist with the development of themes. In addition to the analysis of the data, writing remains a crucial element to thematic analysis, as it informs the complex story of the data in a manner which persuades the reader of the merit and validity of the analysis (Braun & Clarke, 2006). Finally, participants’ direct quotes are included as essential elements to aid the comprehension of key points in the interpretation of the data and to validate the prevalence of themes (Nowell et al., 2017).

### Ethical Considerations

The study was granted ethical approval by the Federation University Human Research Ethics Committee (Reference number A21-173) and was conducted in accordance with the Declaration of Helsinki. Written information regarding the study was provided to participants prior to their consent to participate. If agreeable, all participants provided written consent, while additional verbal consent was received at the time of the interview, which was audio recorded. In case of withdrawing, the participants were requested to inform the interviewer and reassured of no consequences. Participants were also encouraged to inform the interviewer if they became distressed. Psychological support was to be provided, and the national contact number for counseling in Australia was provided.

## Results

From the data collected, four themes emerged and included triggers and help-seeking, accessing formal and informal support, barriers to ongoing support, and relationships, which are discussed in detail.

### Triggers and Help-Seeking

The common triggers of poor mental health among the rural participants were associated with work, family, and environmental factors. Despite these commonalities, the help-seeking behaviors among participants were quite variable and seemingly unique. This was often dependent on their capacity to make decisions at the time to seek help and the insight they had or needed about their condition.

Most participants spoke of being reluctant to seek help. The wife of her acutely unwell husband (Participant 1) reported that the situation was difficult prior to seeking help because she could not understand the changes in her husband’s behavior. In addition, she stated that her husband refused to talk to counselors or psychologists when she had sought support after her husband attempted suicide and continued to have maladaptive coping skills:

He tried to commit suicide and he was just that really withdrawn, angry, stressed, just not being able to handle the usual day to day tasks like showering and making decisions about what to eat. (Participant 1)

This participant’s husband corroborated his wife’s efforts to link him to support services at the time. He ignored this advice due to the state of mind he was in at the time:

I had no coping mechanisms at all. Um, I guess the biggest one was withdrawing, leaving a situation, particularly where there was any contingency plan to do anything else. (Participant 2)

In terms of triggers and help-seeking, the capacity to be self-aware was noted across all three male participants. Collectively, there was a need to realize and conclude that “something” was not right, and signposts existed to assist with this recognition. Participants described being able to identify the issues they experienced and how they intervened by getting support:

Um, I think my wife pointing out to me was a catalyst to me, starting to think about it myself and recognise, “hang on,” you know what you have done. (Participant 2)

Following this, the participant managed to seek the local doctor’s support, and having a plan in place helped him realize there was potentially a “way out” of the situation he was in. This theme embodies triggers and help-seeking where participants emphasized not only identifying issues in their own mental health but also making the next step in seeking help.

### Accessing Formal and Informal Support

Among all participants support was sought from professionals, informal supports, family, or individual networks. Although some participants had access to support at work, there was an overwhelming tendency to not use these services, with one participant stating it was vital to separate personal from his professional life. In this case, most participants sought support from friends or other trusted people with whom they interact with:

This bloke, he comes to help me, but he talks to me. . . He helps me with my tractor, while in ways he’s been there, he’s been the best psychologist that I’ve got. (Participant 3)

Family was also perceived as beneficial where parents, siblings, uncle, and children played an important role in providing support, with families seemingly having a significant level of understanding of where the mental health challenges existed:

Really well actually. Um, they received it really well. I think his mum was kind of, “oh yeah, it runs in the family. You are bound to get it.” (Participant 1)

The participant who preferred to disclose his challenges to his uncle felt a high level of trust, while also not being judged. However, it was key significant others, such as wife, partner, or lovers, who also played a key role in providing support for all male participants. This highlights the importance of family and social networks in providing support for men, while also highlighting that trust, particularly among intimate partners, remains an essential aspect that enables people to open up and be assured a nonjudgmental listening ear is available to help.

Although considered vital, professional support was cited by participants as somewhat problematic due to the inability to access services in rural or regional areas. The challenges included difficulties getting appointments with General Practitioners and Allied Health professionals, such as a psychologist or counselor. In addition, access was also centered on the availability or proximity to services, but more importantly, the associated length of time required to wait before a professional appointment could be achieved. For all, securing an appointment was a lengthy and drawn-out process. One participant clearly stated:

Having access to services and can be quite difficult at times, lots of travelling (two-hour drive) for doctor’s appointments and those sorts of things. (Participant 2)

Participants reported extended periods of time before being able to access a GP appointment. One participant stating *When you need the service, it’s not available* (Participant 4). Similarly, accessing locum GP services was also considered problematic given the need to re-tell and even re-live the experiences for their fleeting visits, where the next visit may mean a new doctor and needing to re-tell and re-live the experience again. Another challenge described by participants was the short length of consultation time which restricted each participant’s capacity to truly express their concerns.

Despite the challenges, there were also benefits regarding accessing professional support being reported, such as the ease of having online or telehealth support or employee assistance helpline, particularly in times of crises when no one else was perceived to be available. When an emergency, such as a suicide attempt, presented itself, participants indicated that the local GPs would make themselves available:

Well, we had a situation where it was an emergency, um another time when he was attempting suicide . . . so we got in straight away. (Participant 1)

This theme brought out the importance of family and key social networks in providing support for men, particularly when services were not accessible. Trust was identified as a vital aspect of this theme, as it enabled men to open up to people they trusted, who they felt would listen and be of genuine help to them.

### Barriers to Ongoing Support

In addition to accessing support, barrier to ongoing support access itself was a major theme that permeated participant discussion. Key challenges included physical access to medication, travel time to the nearest city, and misconception of what the professional services would offer were discussed. Participants stated that medication was not at times readily available at the rural pharmacy and often needed to be ordered. In some cases, participants preferred to utilize the pharmacy and General Practitioner in a distant town to reduce being stigmatized in their town due to their mental health. Travel became challenging due to work commitments, time, and distance. Alternatives such as utilizing telehealth were available to alleviate some of the fear of the community knowing the participants' health concerns:

I still didn’t feel comfortable seeing a doctor in this community. I still don’t get my scripts filled at the pharmacy here. Again, I know some of the people who work at the pharmacy, and I don’t want them to know what I’m dealing with. (Participant 2)

In addition to physical access to services, psychological access was also highlighted as a barrier. Participant 2 felt there was stigma related to mental health within small rural communities, and this greatly limited him to accessing support services locally:

Um, thought there’s a bit of stigma around mental health, um, and being a small community. I didn’t really want everyone to know, you know, “Frank (pseudonym) has a mental health issue, you know.” (Participant 2)

His wife gave an account of how the locum doctor’s attitude toward her husband was challenging and as though the root cause was her husband. In this case, it was felt the doctor was not really listening to his needs:

[The mental health issue] was brought up with a locum and he was horrible. I actually went to that appointment. [The Doctor said] “You just have to lose weight . . .” It was like, “oh, you just got to lose weight . . .” [the doctor was] just tick a box, then out of the door and not my responsibility anymore. (Participant 1)

This theme captured key challenges such as physical access to medication, travel time to the nearest city, and misconceptions of what professional services would and should offer. Some of these factors were centered on to fear and stigma, while also highlighting dismissive professional services.

### Relationships

In terms of relationships, each participant gave an account of how their relationships were impacted by the mental health challenges being experienced. Some participants thought of leaving their relationships which had a negative impact on their significant others. One participant described how difficult it was to understand her husband’s behavioral changes within their marriage and exemplifies the experiences of others:

It was pretty tough. I didn’t quite understand what was going on. It was really hard because he wanted to leave the relationship. (Participant 1)

This same participant also described how she felt when she tried to support her husband, when she states that she needed to be resilient:

Yeah, it’s really tough. It’s really, it’s really hard. I think you have to, because it can be quite degrading toward you and you’ve really got to be in the right mindset, but also not to take it to heart. (Participant 1)

In addition, another participant expressed worry and stated that he might be damaging his partner’s mental health and invading her personal space with his concerns. Children were also reported to have been impacted by the change in behaviors when poor mental health occurred among the male participants. However, some children were reported to be too young to understand what was going on while others were described as having internalized their emotions:

The oldest child was affected much longer. She was quite hurt and she’s quite an anxious person. (Participant 1)

Despite these challenges at the time, a positive outcome of help-seeking for this participant was that his relationship with his children had improved. For men, relationships were a catalyst to developing insight, realizing the need for help, which in turn then improved their personal relationships.

## Discussion

The data consistently demonstrates triggers that lead to the deterioration of an individual’s mental health. Also leads to how and when they seek help for their unique situation. Employment as a trigger of poor mental health has been identified elsewhere within the literature, with rural and remote populations being particularly vulnerable (Kaukiainen & Kõlves, 2020; Seidler et al., 2022). Among rural or regional men, Kaukiainen and Kõlves (2020) indicated that working long hours in industries such as fishery and agriculture, where there is access to lethal means, contribute to increased suicide rates across rural and remote areas of Australia. In addition, natural events or extreme weather changes have also been identified as stressful and triggering contributed to a decline in mental health, leading to suicidal ideation (Kaukiainen & Kõlves, 2020; Trail et al., 2021). A study by Cianconi et al. (2020) reported that suicidal thoughts, sleep disturbance, depression, and anxiety were distressing symptoms resulting from climate change; however, the climate or extreme weather events were not raised in any detail among the participants. Although the above-mentioned are triggers, they can also be considered as effects of mental health experiences. According to Clarke and van Amerom (2008), men tend to avoid personal responsibility for their health and feelings but put the blame on their biology or external forces such as politics.

Seidler et al. (2022) have stated that family stress, relationship breakdown, and first-time fatherhood were situational challenges affecting men’s mental health. In addition, working long hours leading to time apart from strong ties with family increased vulnerability to men’s well-being (Brew et al., 2016). In the current study, co-morbidities, such as neurological issues and cancer, were identified as participant’s deteriorating mental health. Comparing rural to urban Australia, those living with mental illness in rural areas are three times more likely to be at risk of early death due to various physical conditions (Roberts et al., 2018). Help-seeking within this study was dependent on capacity of the individual at that time and their insight into their current acute need, and a few barriers were identified. Barriers include a lack of information regarding available services, doubts about privacy and confidentiality, negative experiences of health services, stigma, and isolation (Kaukiainen & Kõlves, 2020; Labra et al., 2019; Trail et al., 2021). There were also enablers of help-seeking, which were reinforced by Labra et al. (2019), who indicated that a spouse or women, in particular, play a crucial role in assisting men to decide when to seek help.

In addition to the influential impact of a spouse or female loved one, the study highlighted all participants received support from either professional support and/or informal supports, which included friends, family, or social networks, and religion. Gorman et al. (2012) claimed that a relationship with a religious community encourages a deeper connection, further than what professional therapy provides, and such avenues also provide a self-exploration and greater conceptual understanding related to meaning of one’s life, particularly among rural men who experience mental health challenges in rural areas. Although Lucchetti et al. (2021) agreed with positive outcomes resulting from religious spiritual coping, it was also suggested that negative religious coping like feeling punished by God can also result. Furthermore, neurosis and hysteria were considered the worst outcomes of religiosity and spirituality (Lucchetti et al., 2021). However, patients were reported to have some discomfort discussing their spirituality with health professionals; therefore, it is important to explore a better approach to improve interventions (Snider & McPhedran, 2014).

Trail et al. (2021) reported that psychological distress for rural men can be minimized by an increased sense of community and social support structures. Terhaag et al. (2020), suggested that men who lacked close family or friends were at double the risk of suicidal ideation. Whereas Lynne Wilson et al. (2012) argued that the more the family provides care for men with mental health issues, the more their own psychological well-being may be at risk of declining. It is vital to have support in place for both the individual and the family who are supporting their loved one.

Beyond personal support being available, participants cited challenges associated with getting professional support. This often included difficulties in securing an appointment with a General Practitioner or Allied Health Professional, such as a psychologist or counselor. Perkins et al. (2013) suggested that the first choice for seeking mental health support in rural areas is General Practitioners who are in short supply to secure appointments. To reinforce this, wait times were highlighted to be a concern or even a roadblock to seeking help in rural areas (Hull et al., 2017; Perkins et al., 2013). In addition, Perkins et al. (2013) highlighted it is challenging to recruit and retain health professionals, particularly doctors, in rural or remote areas of Australia, which leads to increased wait times. Being able to provide health professionals to support men with mental health issues in rural areas remains to be explored in greater detail within the literature, suggesting additional research needs to occur in this space.

In addition to accessing professional support, participants reported other challenges associated with access, which include physical access to medication, travel time to the nearest city, and misconception of what the professional services would offer. The difficulties include but are not limited to inadequate workforce, long distances, high cost, lack of locally available services, and inadequate infrastructure. It was suggested that providing a variety of health care options, in addition to General Practitioners, would be a means of establishing preventive conversations with men. As such, this may increase health care access, particularly when stigma is perceived as a real or actual challenge among men living in rural communities (Schlichthorst et al., 2016). Ab Aziz et al. (2022) reported that men’s limited consultations with the health care providers are because the health care system is not customized to their needs and lack of men’s health promotions in comparison to women’s health. To improve men’s help-seeking behavior, Ab Aziz et al. (2022) detailed the importance to cater for men’s health through policy change and providing a friendly physical environment that is confidential, convenient, and comfortable.

Despite the challenge of stigma and anonymity being of concern among rural men, online and telehealth as an alternative was considered a vital support when utilized by participants, particularly when experiencing a personal crisis. This finding was supported by Chatterton et al. (2022), who highlighted telehealth was beneficial to rural health consumers, as it enables them to receive care in their homes, while providing increased ease of ongoing assessments and support. During the COVID-19 pandemic, it was challenging for men’s health service providers to meet the needs of men (Shravankumar et al., 2020). Although benefits such as limited time to travel to access support, challenges such as trust, rapport, missing cues like body language, and a reluctance among men to change were related to reduced use of telehealth and helplines (Bradford et al., 2015; Trail et al., 2022). These challenges were reported to be faced by vulnerable groups such as the Indigenous and older people (Shravankumar et al., 2020). However, education and awareness of telehealth as a suitable alternative should be promoted to improve the use of telehealth, rapport, and acceptance and minimize challenges such as anxiety, reluctance, and travel.

The experiences explored throughout this study highlighted that individuals impacted beyond the individual themselves, including married or romantic partners and children. Lawn and McMahon (2014) outlined spouses who care for their partner’s mental health strive to accommodate the condition as well as protect their relationship. For men, the perceived failure to provide for the needs of their family has been recognized to lead to men being emotionally disconnected from their loved ones due to a feeling of failing to measure up to others (Drioli-Phillips et al., 2021). Within these same contexts, children may be at risk of lacking core attachment needs such as emotional and physical nurturing, love, and security, which has implications for their adult mental outcomes, for example, distress (Kamis, 2020). In these circumstances, prolonged exposure to parents’ mental illness may pose greater distress in children progressing to adulthood (Kamis, 2020). McCabe et al. (2015), reported that rural men, including Indigenous men, considered the support of their family to be important in shaping and sustaining positive health behaviors. Conversely, Seidler et al. (2022) postulated that restrictive gender socialization among males has a propensity to lead them to rely entirely on their romantic partner for emotional and social support. It is important for men to maintain relationships that promote continuous support and improve their mental health outcomes (McCabe et al., 2015; Oliffe et al., 2022).

Despite repeated attempts to recruit via social media (Facebook and LinkedIn) as well as word of mouth, we could only recruit four participants. Although phenomenological research does not predetermine a required number of participants, this sample size could only be considered as indicative rather than generalizable. The challenge of recruitment further highlights ongoing stigma regarding mental health among mend and may be why there was a lack of desire to participate in the study. Even though this is a limitation, it enabled a deeper engagement with the participants.

Future research may consider more targeted recruitment methods that address the stigma associated with or perceived by men concerning mental health. Finally, having only one partner or significant other who was willing to participate minimized the rich accounts of what may be observed among men through their loved one’s journey. Future research may need to consider incentives for participants to promote greater numbers of participants to be recruited or other avenues that reduce perceived stigma, safe spaces to speak up, or even key ambassadors to enable a greater number of men to want to talk.

## Conclusion

The findings of this study identified four themes, which encompassed triggers and help-seeking, Support, Access, and Relationships. When triggers occur, men are often supported by their significant others, family, friends, or other people in the community to seek help. However, it is not until these issues impact the lives of the families and/or significant others that help is typically sought. Our study has found that even when men are psychologically prepared to get help, it can be difficult to access. Overall, improving access goes beyond more doctors and specialists in rural areas, and instead must focus on supporting families and loved ones to recognize when there may be a mental health issue. Providing services in rural areas can be problematic, therefore, to address issues of access, provide a variety of services including telehealth, online forums, and meet-up groups within the community. Finally, mental health and promotion should be encouraged in rural and remote areas to promote understanding, minimize stigma, and improve access to support. Although the study met its objectives, a number of unanswered questions remain highlighting the need for future research.
